# A new species of rake-legged mite, *Caeculus cassiopeiae* (Prostigmata, Caeculidae), from Canada and a systematic analysis of its genus

**DOI:** 10.3897/zookeys.926.48741

**Published:** 2020-04-13

**Authors:** Jared Bernard, Lisa M. Lumley, Matthias Buck, Tyler P. Cobb

**Affiliations:** 1 Invertebrate Zoology, Royal Alberta Museum, 9810 103A Avenue NW, Edmonton, Alberta T5J 0G2, Canada Royal Alberta Museum Edmonton Canada; 2 Plant & Environmental Protection Sciences, University of Hawaii-Mānoa, 3050 Maile Way, Honolulu, HI 96822, USA University of Hawaii-Mānoa Honolulu United States of America

**Keywords:** Acari, character state matrix, comparative morphology, phylogeny, Trombidiformes

## Abstract

The genus *Caeculus* Dufour (Prostigmata, Caeculidae) contains 19 previously described species, most of which are found in North America, and for which no comprehensive phylogenetic treatment exists. Here, one new species from Alberta, Canada, is described: *Caeculus cassiopeiae* Bernard & Lumley, **sp. nov.**, and another caeculid known to be present in Canada is documented. The new species is characterized within the genus with a character state matrix, from which an updated key is produced. A systematic analysis of all 20 species based on morphological and geographical distribution traits obtained from literature represents the first phylogenetic review of the genus.

## Introduction

The Caeculidae contains 108 previously described species of large (750–3000 µm) prostigmatic mites in seven genera distributed worldwide, with 19 species in the genus *Caeculus* Dufour (Walter et al. 2009; Taylor et al. 2013; Mangová et al. 2014; Ott and Ott 2014; Taylor 2014; Fuangarworn and Butcher 2015; Rivas et al. 2016; Per et al. 2017; Porta et al. 2019). Employing the spiniform setae on legs I for which they are called rake-legged mites, caeculids are ambush predators of small arthropods including collembolans (Otto 1993). They do so camouflaged against rocky or sandy substrates in arid environments (Coineau 1974). Other than morphological reviews by Vitzthum (1933), Grandjean (1944), and Coineau (1974), a dichotomous key by Franz (1952), and original species descriptions, Caeculidae have not been widely collected or studied. Hence, a phylogenetic assessment of *Caeculus* has not yet been attempted.

In July 2014, we collected two specimens in yellow pan traps in Medicine Hat, Alberta, Canada. They represent a new species, *Caeculus cassiopeiae* sp. nov., described below. This record increases the number of known Canadian caeculids to two species. Evert E. Lindquist (Canadian National Collection, Ottawa, Ontario, Canada) collected the other species in Alberta’s Writing-on-Stone Provincial Park in 1978, and the same species in Alberta’s Waterton Lakes National Park in 1980, which are the only previously known collections from Canada and are deposited in the CNCI. He identified this species only to *Caeculus* and did not publish it, but herein we identify it as *C. cremnicolus* Enns. The only published record of caeculoids in Canada is by Lindquist et al. (1979), who listed at least one unidentified caeculid species in southern inland British Columbia based on personal communication with Valin G. Marshall (Canadian Forest Service, Victoria, British Columbia, Canada), but without collection or further identification information. Although Lindquist et al. (1979) estimated the potential discovery of 2 additional undetected species based on records in the United States, the updated catalogue of Canadian Acari still lists only one previously recorded caeculid species (Beaulieu et al. 2019).

We constructed a character state matrix to compare *C. cassiopeiae* sp. nov. to the descriptions of all other known species of *Caeculus*. In the absence of molecular data, we used the matrix as a phenotypic platform for a phylogenetic analysis of the genus, which illuminates the congeners most closely related to the new species, and provides a springboard for further assessment of the genus.

## Materials and methods

On 27 July 2014 in Medicine Hat, Alberta, Canada, we collected two female caeculid specimens in yellow pan traps filled with soapy water (Marshall et al. 1994), which for two days were placed on both the arid SW slope of a coulee (glacially formed from sandstone and clay) and on the adjacent plain dominated by non-native crested wheat grass (*Agropyron cristatum* (L.) Gaertner) and alfalfa (*Medicago sativa* L.). We revisited the same locality on June 26, 2017 and using a paintbrush collected additional specimens, which were found only on open, exposed soil surfaces of the arid SW slope during the hottest part of the day (13:45–15:30, ≥ 32 °C). The soil surfaces had a thin hardened crust, possibly created by drying after rainfall.

According to the Köppen-Geiger climate classification system, Medicine Hat is a cold semi-arid steppe (*BSk*) (Peel et al. 2007) with a mean annual precipitation of 322.6 mm and a mean annual temperature of 6.1 °C (NCDIA 2017). Based on geological maps of the region (Berg and McPherson 2005) and the presence of well-rounded gravel and fine-grained sediment, the surficial geology is consistent with Quaternary alluvium. Medicine Hat furthermore has brown chernozem soil by the Canadian classification (Fuller 2010; NSBD 2017), synonymous with ustic mollisol in the USDA soil taxonomy (Haynes 1998).

After photographing the specimens collected in 2014 with a K2 DistaMax long-distance microscope (Infinity Photo-Optical, Boulder, Colorado, USA), we stored one in 95% ethyl alcohol (EtOH) and cleared the other in 85% lactic acid (Thermo Fisher Scientific, Waltham, Massachusetts, USA) and dissected it before mounting on a slide in a solution of 1.66 g polyvinyl alcohol, 10 mL lactic acid, 1 mL glycerol, and 10 mL distilled water (produced by Bioquip, Rancho Dominguez, California, USA) for analysis under dissection and compound microscopes, both of which contributed to creating the free-hand illustrations. With the 2017 specimens, we kept two alive for observation and stored the remainder in 95% EtOH.

In describing the idiosomal morphology of the new species, we followed the terminology outlined by Coineau (1974), which is based on the model of idiosomal divisions by Grandjean (1969) and other aspects of caeculid morphology described by Grandjean (1944). Notation for setae follows Coineau (1964, 1967a, b, 1969, 1974). As described by Coineau (1964), the eponymous characteristic of rake-legged mites is their elongated thickened spiniform leg setae, which are known as “rake” setae and are labelled as such herein.

To compare our specimens to other congeners using established criteria, we mined morphological and geographical distribution data from all known publications on *Caeculus* to construct a standard categorical character state matrix of the female of 23 taxa in Mesquite version 3.2 (Maddison and Maddison 2017), including all 20 species of *Caeculus* and three species of *Neocaeculus* Coineau (Table 1). We incorporated additional chaetotactic data into the matrix for *C. echinipes* Dufour from Jacot (1936), and for both it and *C. americanus* Banks from Coineau (1974). The resulting matrix includes mostly characters that are clearly described and/or illustrated in the species descriptions. Missing data are denoted with a “?” and uncertainty between states is characterized with a “/”, following the notation used by Maddison and Maddison (2017). In rare circumstances we inferred characters that were consistently mentioned in the descriptions. For instance, because Mulaik (1945) noted tarsal bothridial setae for some species but not for others, we reasoned that he would mention the trait if present; thus if illustrations or text did not include a trait that had been described in other species by the same author, we treated the trait as absent. The matrix did not include information on ecology or internal anatomy because this was lacking in publications.

We conducted a parsimony analysis of these phenotypes with PAUP* version 4.0β10 (Swofford 2000), which involved a heuristic search with a tree-bisection-reconstruction branch-swapping algorithm for 5000 replicates. If the maximum branch length was zero, we set branches to collapse. The setation of *N. imperfectus* Taylor, Gunawardene & Kinnear resembles that described by Coineau (1974) as the holotrichous setal complement of Caeculidae, so we designated this species as the outgroup (but not the other *Neocaeculus* species) for our phylogenetic analysis. Note that the ingroup of this study includes only species currently in the genus *Caeculus* and excludes those that have been reassigned to other genera (reviewed in Taylor et al. 2013). We visualized the majority consensus tree with FigTree 1.4.3 (Rambaut 2012).

**Table 1.** Standard categorical character state matrix for *Caeculus* females, as well as three outgroup taxa, used for cladistic analysis. Polymorphism is denoted by “&”, uncertainty by “/”, missing data by “?”, and inapplicable data by “-”. “†” signifies a size character not included in phylogenetic assessment.

Taxon	1	2	3	4	5	6	7	8	9	10	11	12	13	14	15	16	17	18	19	20	21	22	23	24	25	26	27	28	29	30	31	32	33	34	35	36	37	38	39	40	41	42	43	44	45	46	47	48	49	50	51†
*Caeculus americanus* Banks, 1899	2	2	1	2	0	0	0	0	1	0	0	1	1	0	0	1	0&1&2	0&1	0&1&2	0&1	1&2	0&1	?	?	?	?	?	1	0	?	0	0	0	0/1	2	0/1	3	2	?	2	0	3	?	?	?	?	1	0	0	1	2
*Caeculus archeri* Mulaik, 1945	1	1	0	1	0	0	0	0	0	1	0	0	1	1	1	1	2	0	2	0	0	1	?	3&4	3	?	?	0	0	0	0	0	0	0	2	1	1	3	0	?	0	?	0	0	1	1	0	0	0	1	2
*Caeculus calechius* Mulaik, 1945	2	0/1	0	1	0	0	0	0	0	0	0	0	0	0	0	1	1	1	0	0	0	1	?	3	3	?	?	0	0	0	0	0	0	?	1	3	1	2	?	0	0	3	0	0	0	1	0	0	0	1	2
*Caeculus cassiopeiae* Bernard & Lumley, sp. nov.	3	2	0&1	2	0	0	0	0	1	1	0&1	0&1	1	0&1	1	1	2	0&1	2	1	1	1	6	4	2	1	1	2	0	1&2	0	0	0	1	2	1	3	4	0	3	0	2&3	0	0	1	1	0	0	0	1	2
*Caeculus clavatus* Banks, 1905	1	1	?	?	0	0	?	?	?	?	?	?	?	?	?	1	?	?	?	?	?	?	?	?	?	?	?	0	0	2	1	0	1	0	1	1	1	2	0	?	0	?	?	?	?	?	0	0	0	1	1
*Caeculus cremnicolus* Enns, 1958	3	1	1	2	0	0	0	0	0	0&1	0	1	1&2	1	0&1	0	1&2	0&1	2	1	0	1	2	4	2	0	0	0	0	0	0	0	0	1	1	1	0	1&2	0	3	0	0	0	0	1	1	1	0	0	1	2&3
*Caeculus crossleyi* Hagan, 1985	1	1	1	3	0	0	0	1	1	0	1	0	2	2	1&2	1	3	0	5	0	0	1	0	4	3	1	0	0	0	0&1	0	0	0	0	1	1	0	1/2	0	3	0	0	1	1	1	1	1	0	0	1	0
*Caeculus dorotheae* Mulaik, 1945	2	3	0	0	0	0	0	0	0	0	0	0	0	0	0	1	1	0	2	0	0	0	?	?	?	?	?	2	0	3	0	0	0	?	2	?	4	5	?	3	0	3	0	0	0	0	0	1	0	1	1&2
*Caeculus echinipes* Dufour, 1832	0	1	1	2	0	0	0	0&1	0	0&1	0&1	0&1	1	1	0&1	1	1&2	0&1	3	0	1	0	4	3	2	0	0	0	0	0	0	0	0	1	2	2	1	3	0	1	0	?	1	1	1	1	1	0	0	1	3
*Caeculus gertschi* Mulaik, 1945	2	1	1	1	0	0	0	0	0	0	0	0	0	0	0	1	1	1	2	0	0	1	5	2	3	1	0	1	1	1	0	0	0	?	2	?	2	?	?	1	0	1	0/1	0/1	0/1	0/1	0	0	0	1	1
*Caeculus hardyi* Mulaik & Allred, 1954	2	1	2	1	0	0	0	0	0	0	0	0	0	0	0	1	1	0	1	1	1	1	?	?	?	?	0	1	0	0	0	0	0	0	1	0	1	0	1	?	0	?	0	0	0	0	0	0	0	1	1&2
*Caeculus hypopachus* Mulaik, 1945	2	1	0	1	1	0	0	0	0	0	0	0	0	0	0	1	1	1	0	0	0	0	?	?	?	?	0	1	1	1	0	0	0	?	1	?	2	?	?	?	0	?	0	0	0	0	1	0	0	1	1
*Caeculus janetae* Higgins & Mulaik, 1957	2	1	1	1	0	0	0	0	0	0	0	0	1	0	0	1	1	0&1	2	0	0	1	5	2&3&4&5	2&3	1	0	0	0	1	0	0	0	0	3	0	4	0	0	?	0	?	0	0	0	0	0	0	0	1	1&2
*Caeculus kerrulius* Mulaik, 1945	2	0	0	1	0	1	0	0	0	0	0	0	0	0	1	0	2	0	2	1	1	0	?	?	?	?	0	0	0	2	0	0	0	0	2	0	1	3	0	1	1	0	0	0	1	1	1	0	0	1	1
*Caeculus krantzi* Coineau, 1974	4	1	1	2	0	0	0	1	0	1	1	0	1	1	1	1	2	0	4	1	0	1	4	3	2	0	0	?	0	?	?	?	?	?	?	?	?	?	?	?	0	?	?	?	?	?	0	0	0	1	3
*Caeculus lewisi* McDaniel & Boe, 1990	3	3	0	2	0	0	0	0	0&1	0&1	0	0&1	0	0	0&1	1	1&2	0&1	3	1	0	1	5	3&4	2	2	1	2	0	1&2	0	0	0	0	2	0&1	2&3	3&4	0	0	0	1&2	0	0	1	1	0	0	0	1	1&2
*Caeculus mariae* Higgins & Mulaik, 1957	4	1	1	1	0	0	0	1	0	1	0	1	1	1	0	1	1	0	2	0	0	1	?	?	?	?	0	0	0	0	0	0	0	?	1	?	1	?	2	2	0	0	0	0	0	0	0	0	0	1	0
*Caeculus pettiti* Nevin, 1943	1	1	1	1	0	0	0	0	0&1	0&1	0	0&1	1&2	1	1&2	0	3	0&1	2&3	0&1	0	1	5	4	2	2	1	2	0	0	0	0	0	0	1	1	1	3	0	3	0	0	0	0	0	0	1	0	0	1	2
*Caeculus tipus* Mulaik, 1945	2	2	0&1	1&2	0	0	0	0	1	0	0&1	0&1	1	0	1	1	1&2	0&1	0&1&2&3	0&1	0	1	1	0&1&2	2	1	0	1	0	1	0	0	0	0	1	1	2	4	3	2	0	2	0	0	1	1	0	0	0	1	2
*Caeculus valverdius* Mulaik, 1945	2	2	0&1	2	0	0	0	0	2	0	0	0	1	1	0	1	2	0	1	1	0	1	?	3	3	?	0	2	0	2	0	0	0	?	2	4	3	4	?	2	0	2	0	0	1	1	0	0	0	1	2
*Neocaeculus imperfectus* Taylor et al., 2013	5	1	1	0	0	0	0	0	0	0	0	0	0	0	0	0	1	0	0	0	0	1	3	3	1	2	0	0	1	4	0	-	-	1	2	3	1	3	0	2	1	3	0	0	1	1	0	0	1	0	1
*Neocaeculus kinnearae* Taylor, 2014	5	1	1	0	1	0	0	0	0	0	0	0	0	0	0	0	1	0	1	1	1	1	3	2	1	1	0	3	0	5	1	–	-	0	1&2	1	1	4	0	2	0	2	0	0	1	1	0	0	1	0	1
*Neocaeculus nudonates* Taylor, 2014	5	1	1	0	0	0	0	0	0	0	0	0	0	0	0	0	1	1	0	0	1	1	4	3	2	1	0	0	0	1	1	-	-	1	1	1	0	2	0	1	0	0	0	0	1	1	1	0	1	0	0

### Repositories (see Zhang 2018 for abbreviations)

**CNCI**Canadian National Collection of Insects, Arachnids and Nematodes, Ottawa, Ontario, Canada;

**PMAE**Royal Alberta Museum (formerly Provincial Museum of Alberta) Invertebrate Zoology Collection, Edmonton, Alberta, Canada.

### Abbreviations of morphological characters

**AD** adanal sclerite;

**AG** aggenital sclerite;

**CH** chelicera;

**IL** idiosomal length;

**IW** idiosomal width;

**PA** palp;

PG progenital valve;

**PS** pseudanal sclerite.

#### Caeculus cassiopeiae

Taxon classificationAnimaliaTrombidiformesCaeculidae

Bernard & Lumley sp. nov.

DE2AB6DE-B683-5DCF-ADA4-A1678DF6EAA5

http://zoobank.org/BC876F53-B89C-4AEA-ABC7-EA8CA18B7F91

**Figure 1.**
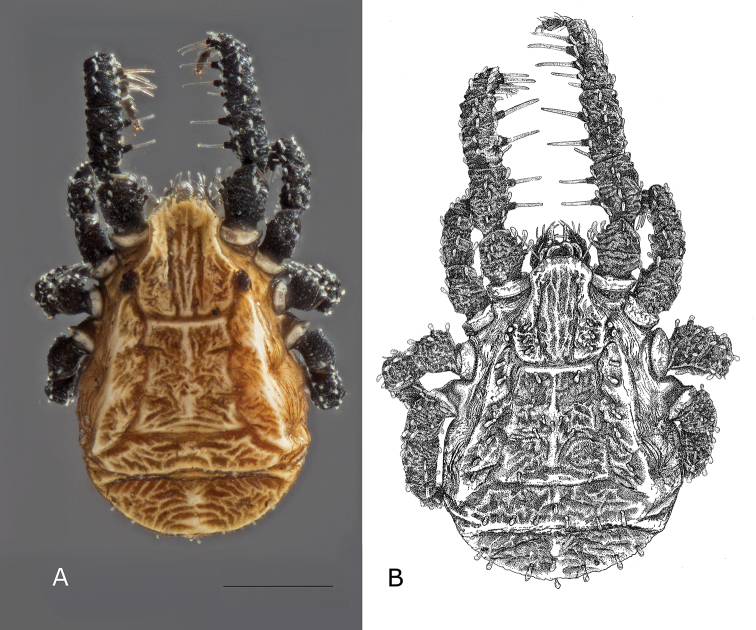
*Caeculus cassiopeiae* sp. nov., female **A** micrograph showing coloration **B** habitus illustrating cerotegument texture. Scale bar: 0.5 mm.

**Figure 2.**
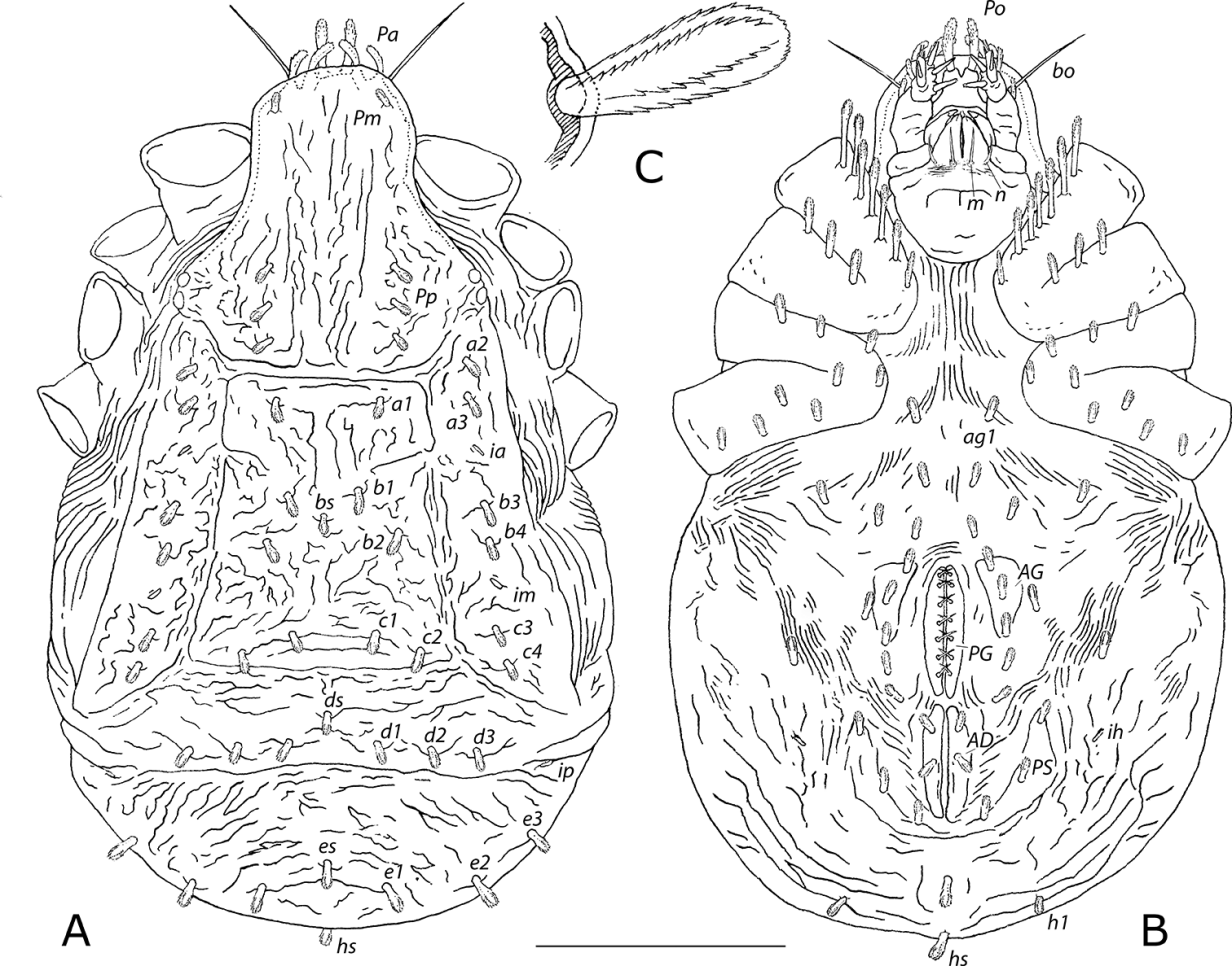
*Caeculus cassiopeiae* sp. nov., female idiosoma **A** dorsum **B** venter; *PG*, progenital valve; *AG*, aggenital sclerite; *AD*, adanal sclerite; *PS*, pseudanal sclerite **C** detail of *bs* seta, exemplifying typical clavate idiosomal seta. Scale bar: 0.5 mm (**A, B**).

**Figure 3.**
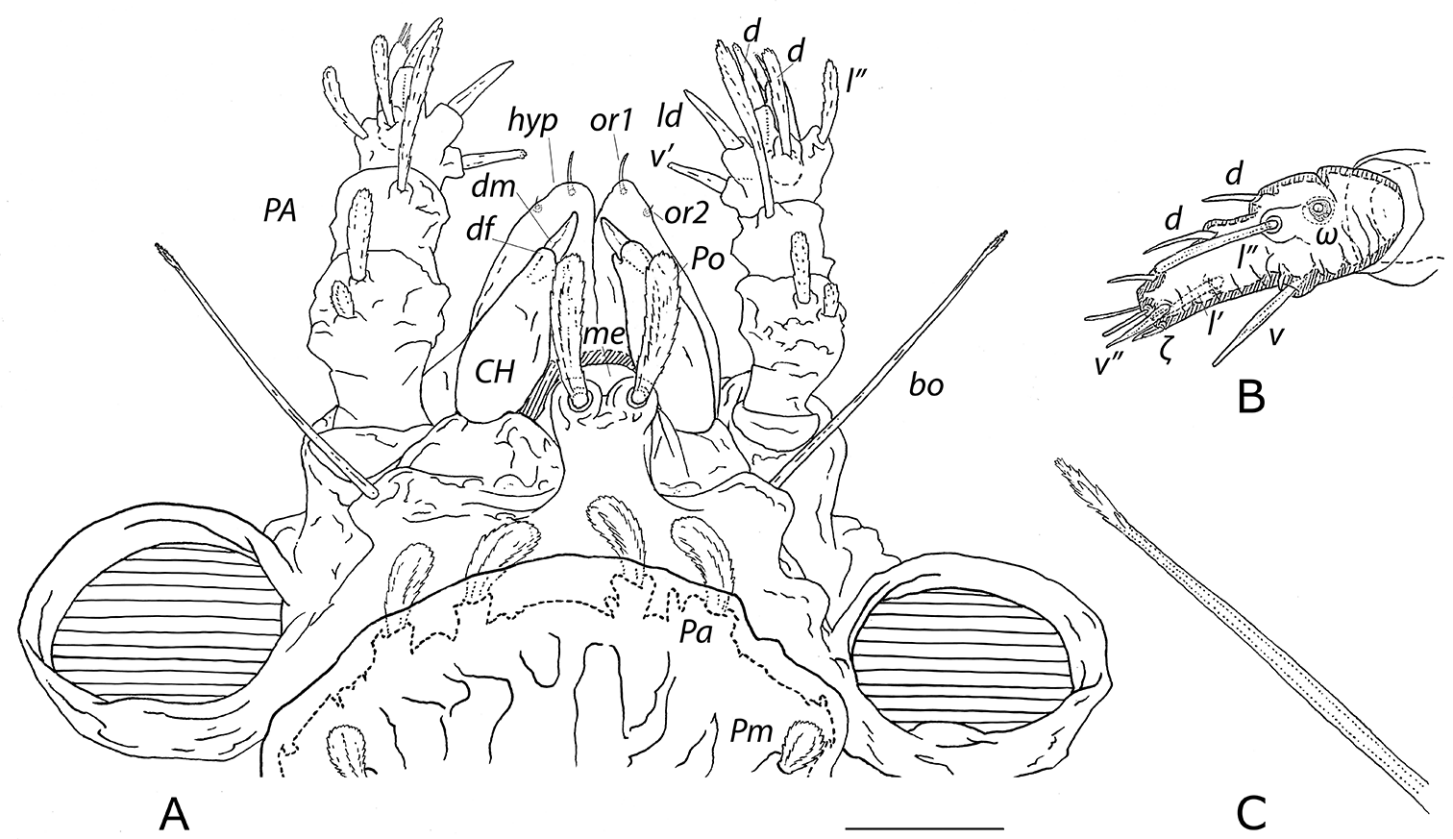
*Caeculus cassiopeiae* sp. nov., female **A** anterodorsal view of rostrum and gnathosoma **B** detail of palp tarsus **C** detail of distal bothridial seta. Abbreviations: *CH*, chelicera; *PA*, palp; *bo*, bothridial seta; *d*, dorsal; *df*, reduced fixed cheliceral digit; *dm*, movable cheliceral digit; *ζ*, eupathidium; *hyp*, hypostome; *l*”, posterolateral; *ld*, laterodorsal; *me*, median eye; *Po*, naso seta; *ω*, solenidion; *v*’, anteroventral; *v*, ventral; *v*”, posteroventral. Scale bar: 0.1 mm (**A**).

**Figure 4.**
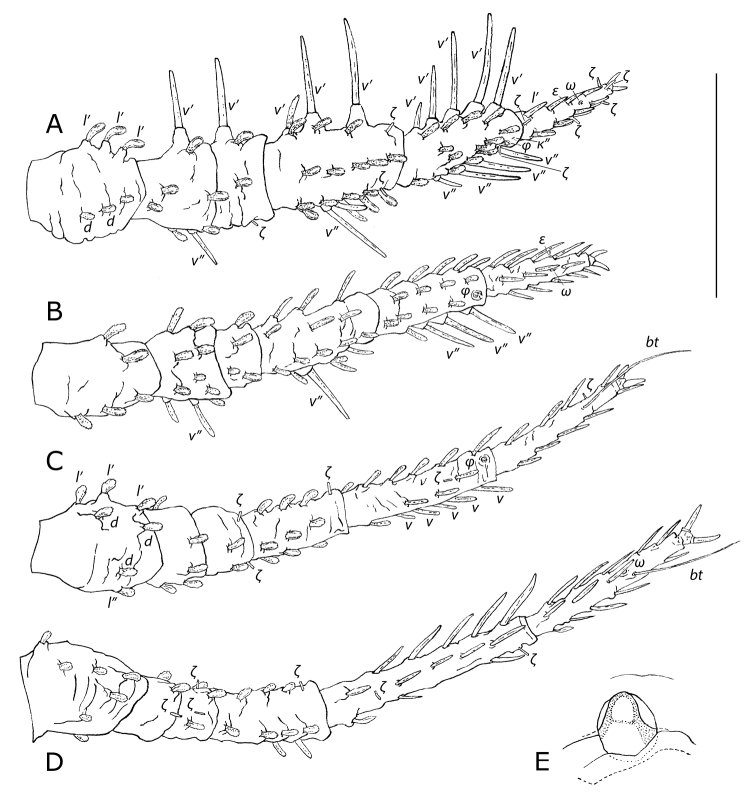
*Caeculus cassiopeiae* sp. nov., female **A–D** dorsal view of legs I–IV **E** detail of solenidion *ω* on tarsus I. Scale bar: 0.5 mm (**A–D**). Abbreviations: *ε*, famulus; *ζ*, eupathidium; *κ*”, microseta; *φ*, tibial solenidion; *ω*, tarsal solenidion; *bt*, tarsal bothridial seta; *d*, dorsal; *l*’, anterolateral; *l*”, posterolateral; *v*’, anteroventral; *v*, ventral; *v*”, posteroventral.

Figures 1 , 2 , 3 , 4

##### Material examined.

***Holotype*.** Canada • 1 ♀; Alberta, Medicine Hat, near Gas City Campground; 50°2.23'N, 110°43.56'W; elev. ca 700 m; 26–27 Jul. 2014; M. Buck leg.; yellow pan traps; arid SW slope and adjacent disturbed plain of *Agropyron cristatum* and *Medicago sativa*; det. J. Bernard and L. Lumley, 30 Aug. 2016; cleared in 85% lactic acid, dissected, and slide-mounted; PMAE M00019466.

***Paratype*.** Canada • 1 ♀; ibid.; stored in 95% EtOH; PMAE M00030967.

##### Other material.

Canada • 8 ♀♀; ibid.; 26 Jun. 2017; L. Lumley leg.; collected with paintbrush; hard dry soil surface; det. L. Lumley; stored in 95% EtOH; PMAE M00030972 to M00030979.

##### Diagnosis.

The five *b* setae on the centrodorsal opisthosoma are arranged in an M, three pairs of *Pp* setae are aligned on the posterior third of the aspidosoma, and trochanter III has three setae along anterolateral surface.

##### Female description

(*N* = 2, all measurements in micrometres, μm).

***Idiosoma dorsum*** (Figs 1–3). 1380–1592 = idiosomal length (*IL*); 1.920 × longer than greatest width (at level of posterior margin of mediodorsal opisthosomal sclerites) (718–829 = *IW*). Sclerites tawny with pale ridges, ochre to raw sienna between sclerites, translucent white setae (Fig. 1A). All clavate idiosomal setae barbed (Fig. 2C). Rostral region with dark brown anteriorly projecting naso bearing one pair elongated clavate setae *Po* (74–94, ~0.054 × *IL*), one median eye immediately inferior to base of naso (Fig. 3A), one pair long, thickened attenuate bothridial seta *bo* (162–188, ~0.118 × *IL*) with barbed distal end (Fig. 3C), each inserted in anterolaterally projecting bothridium posterolaterad to median eye. Aspidosoma length 485–560 (0.352 × *IL*), width 425–491 (0.592 × *IW*) anterior to eyes, posterior margin of sclerite 309–357 (0.430 × *IW*); pronounced furrow along length of medial axis containing three or four shallow longitudinal reticulated ridges, anterior furrow width 135–157 (~0.189 × *IW*), posterior furrow width 102–118 (~0.142 × *IW*), posterior furrow depth 35–41 (~0.050 × *IW*); two pairs procurved clavate *Pa* setae on anterior margin; none or one pair clavate *Pm* setae near corners of anterior margin; three pairs clavate *Pp* setae medial to eyes on posterior lateral margins, aligned parallel to mid-sagittal plane; area around eyes dark, posterior eyes 1.077 × diameter of anterior eyes (Fig. 2A), holotype with diminutive fourth *Pp* seta anterior to other three on right side. Centrodorsal opisthosomal sclerite trapezoidal, length 425–490 (0.308 × *IL*), posterior margin 477–552 (0.665 × *IW*); one pair clavate *a1* setae near anterior margin of sclerite midway between mid-sagittal plane and lateral margins; 2.5 pairs clavate *b* setae: *b1* pair anterior to mid-transverse plane and third of distance between mid-sagittal plane and lateral margins, *b2* pair posterior to mid-transverse plane and midway between mid-sagittal plane and lateral margins, one unpaired median *bs* seta present between anterior and posterior pairs, the five setae together forming M-shape; one or two pairs clavate *c* setae along posterior margin (sometimes *c1*, always *c2*) (Fig. 2A). Laterodorsal opisthosomal sclerites each bearing five or six clavate setae in tandem, two (*a2* and *a3*) near anterior margin, one or two *b* setae at middle of sclerite (always *b3*, sometimes *b4*), and two *c* setae near posterior margin (*c3* and *c4*); lyrifissure *ia* between *a3* and *b3*; lyrifissure *im* between *b4* and *c3* (Fig. 2A). Mediodorsal opisthosomal sclerites fused, bearing clavate *d1*, *d2*, and *d3* setal pairs, with or without unpaired median clavate *ds* seta slightly anterior to these; posterior opisthosomal sclerite length 0.161 × *IL*, bearing clavate *e1*, *e2*, and *e3* setal pairs and unpaired clavate *es* seta slightly anterior to these; lyrifissure *ip* near lateral margin between mediodorsal and posterior opisthosomal sclerites (Fig. 2A). Pluriposterior accessory sclerite bearing three clavate setae, including one unpaired median clavate *hs* seta (Fig. 2B).

***Idiosoma venter*** (Fig. 2B). Epimeres black to dark brown, cerotegument tawny, progenital valves dark sienna, aggenital sclerites raw sienna, adanal sclerites brown ochre, pseudanal sclerites raw sienna, translucent white setae. Epimeral setal formula (I–IV) 7-3-4-5. Epimeres I and II fused, anterior margin of epimere I bearing seven clavate setae, most proximal seta slightly less expanded than others and setae get progressively longer distally, distalmost ~76–120 (~0.071 × *IL*), epimere II with three clavate setae along anterior margin; epimeres III and IV fused (separate from I + II), epimere III with three clavate setae along anterior margin and one clavate seta midway along proximal margin, epimere IV with one clavate seta at anteroproximal margin, one clavate seta on midline a third epimeral length from proximal margin, and three clavate setae along posterior margin (Fig. 2B). Progenital valves (*PG*) each with seven smooth acuminate simple setae; aggenital sclerites (*AG*) each with three clavate setae; surrounding ventral cuticle bearing nine pairs of clavate setae, including pair *ag1* between epimeres IV (Fig. 2B). Adanal sclerites (*AD*) each bearing two clavate setae; pseudanal sclerites (*PS*) each with three clavate setae; one unpaired medial clavate seta posterior to anus; lyrifissure *ih* laterad to anterior pseudanal sclerite (Fig. 2B).

***Gnathosoma*** (Figs 2B, 3A, B). Palps black, chelicerae dark brown, subcapitulum black to dark sienna, translucent white setae. All clavate setae barbed, all simple setae smooth. Palps (*PA*) five-segmented, setal formula, trochanter-tarsus, solenidia *ω* and eupathidia *ζ* in brackets: 0-2-1-5-10(1*ω*+1*ζ*); trochanter without setae; femur bearing two dorsal clavate setae midway along length, with proximal seta a third length of distal seta; genu bearing longest subclavate seta on tubercle at distal laterodorsal margin; tibia bearing five setae: one proximal anteroventral barbed spiniform seta, one laterodorsal spiniform seta with barbed distal end, one proximal dorsal elongated clavate seta, one distal dorsal barbed spiniform seta, and one posterolateral clavate seta (Fig. 3A); well-developed tarsus (Fig. 3B) bearing three dorsal smooth acuminate spiniform setae, one elongated spiniform seta with barbed distal end midway on ventral surface, one elongated barbed subclavate seta midway along posterolateral surface slightly distal to a minute solenidion *ω* recessed in large receptacle, one anterolateral smooth acuminate spiniform seta a third tarsal distance from distal end, one short smooth acuminate seta at distal posteroventral margin, two simple setae at distal end, and one eupathidium *ζ* posterior to one minute smooth spiniform seta at distal ventral margin. Chelicerae (*CH*) each with fixed digit regressed to lobe and movable digit uncinate (Fig. 3A). Subcapitulum bearing two pairs of simple adoral *or* setae (Fig. 3A) and two pairs of elongated thickened acuminate simple hypostomal setae *m* and *n* along base of hypostome, *m* medial to and slightly longer (53–63, ~0.039 × *IL*) than *n* (Fig. 2B).

***Legs*** (Fig. 4). Black with translucent white setae. All clavate setae barbed, all rake/spiniform setae smooth. Formulae of leg setae (including rake setae), trochanter-tarsus, tarsal bothridial setae *bt*, solenidia *φ*/*ω*, eupathidia *ζ*, microseta *κ*”, and famulus *ε* in brackets: leg I 5/6–8+3(1*ζ*)–21(2*ζ*)–22(1*φ*+1*ζ*+1*κ*”)–12(1*ω*+5*ζ*+1*ε*); leg II 5–10+4–16–17(1*φ*)–14(1*ω*+1*ε*); leg III 7–5+2(2*ζ*)–9(1*ζ*)–15(1*φ*+1*ζ*)–9(1*bt*+1*ζ*); leg IV 7–2(1*ζ*)+3(2*ζ*)–9(1*ζ*)–13(2*ζ*)–13(1*bt*+1*ω*). Leg I length 1301–1502 (~0.943 × *IL*; Fig. 4A); trochanter I bearing three procurved clavate setae on tubercles along anterolateral margin, two or three dorsal clavate setae; basifemur I with one rake seta on anteroventral margin and one rake seta on posteroventral surface; telofemur I with one rake seta on anteroventral margin and one eupathidium *ζ* a third the length from distal end on posteroventral margin; genu I with two anteroventral rake setae, one elongated anteroventral subclavate seta near proximal margin aligned with rake setae, three anterolateral clavate setae, one short anterolateral eupathidium *ζ* near distal margin, five clavate dorsal setae, five posterolateral setae with middle seta elongated subclavate and remainder clavate, one short eupathidium *ζ* near distal posterolateral margin, five posteroventral setae with most proximal clavate, followed by one rake seta and three elongated subclavate setae; tibia I with four anteroventral rake setae, one elongated spiniform seta near proximal anteroventral margin in line with rake setae, four anterolateral clavate setae, five clavate dorsal setae, four posterolateral clavate setae, three posteroventral rake setae, and one spiniform seta on posteroventral surface proximad to rake setae, and the following three near distal anterolateral margin, most distal to least: one microseta *κ*”, one recessed solenidion *φ* in large receptacle, and one eupathidium *ζ*; tarsus I bearing four spiniform setae along each anterolateral, posterolateral, and posteroventral margins, one recessed solenidion *ω* in large receptacle situated a third the tarsal length from distal end on dorsal surface (Fig. 4E), one famulus *ε* midway along anteroventral surface, and eupathidia *ζ* at the following locations: proximal dorsal surface, midway along posterolateral margin, a tenth the tarsal length from distal end on posteroventral margin, at distal anteroventral margin, and at distal anterolateral margin. Remaining clavate setation for leg I as in Fig. 4A. Chaetotaxy of other legs as in Fig. 4B–D. Basifemur II with one elongated barbed subclavate seta midway along posteroventral surface; genu II with one rake seta a third the distance along posteroventral surface; tibia II with three posteroventral rake setae, one dorsal solenidion *φ* near distal margin; tarsus II bearing one recessed solenidion *ω* in large receptacle a third length from distal end on dorsal surface, slightly distal to one anteroventral famulus *ε* (Fig. 4B). Trochanter III with three anterolateral clavate setae and two or three dorsal clavate setae; tibia III with one solenidion *φ* near distal margin on dorsal surface (Fig. 4C). Tarsi III and IV each with one elongated smooth slender bothridial seta *bt* a quarter of the length from distal margin on dorsal surface (125–145 [~0.091 × *IL*] on tarsus III; 143–166 [~0.104 × *IL*] on tarsus IV); tarsus IV with one solenidion *ω* a third the length from distal end on dorsal surface (Fig. 4C, D). Tarsal claws on all legs equal in size.

**Male** and immatures unknown.

##### Etymology.

The *b* setal arrangement on the centrodorsal opisthosoma resembles the five-star constellation named for Cassiopeia, the vain wife of King Cepheus in Greek mythology. The constellation is also known as the “Celestial M” given its orientation to the horizon when it ascends in the night sky on its arc around Polaris, and is known as the “Celestial W” as it sets. Cassiopeia’s rise is most observable in the evenings during northern autumns. The genitive epithet abides by Articles 11.9.1.3 and 31.1.2 of the International Code of Zoological Nomenclature, and hence means “Cassiopeia’s rake-legged mite.”

##### Ethology.

We observed individuals both walking and motionless with legs I positioned in a raptorial manner above the soil surface. A captive individual consumed a prostigmatan we collected from the same exposed soil.

#### Caeculus cremnicolus

Taxon classificationAnimaliaTrombidiformesCaeculidae

Enns, 1958

91D1FE08-0AE8-5BF9-9DD9-0CE7A2E3A4A8

Figure 5

##### Material examined.

Canada • 2 ♀♀, 1 deutonymph, 1 tritonymph; Alberta, Waterton Lakes National Park; 29 Jul. 1980; E.E. Lindquist leg.; under rocks in canyon bottom; det. J. Bernard, 4 Mar. 2015; slide-mounted; CNCI • 1 ♀, 2 deutonymphs, 1 tritonymph; Alberta, Writing-on-Stone Provincial Park; 12 Aug. 1978; E.E. Lindquist leg.; under rocks in hoodoo area [hoodoo = rock column formed by soft sediment eroding under harder sediment]; det. J. Bernard, 4 Mar. 2015; slide-mounted; CNCI.

##### Previously known localities.

USA – Arkansas, Buffalo National River, Boen Gulf and Steel Creek; Petit Jean State Park (Skvarla et al. 2013) – Missouri, Baskett Wildlife Research and Education Area, Devil’s Backbone; Easley; Wilton (holotype) (Enns 1958).

##### Diagnosis.

Distinguished by its unfused mediodorsal opisthosomal sclerites, by its dark sclerites in adult mites, and by three barbed clavate *b* setae on the centrodorsal opisthosoma arranged in a triangle.

**Figure 5.**
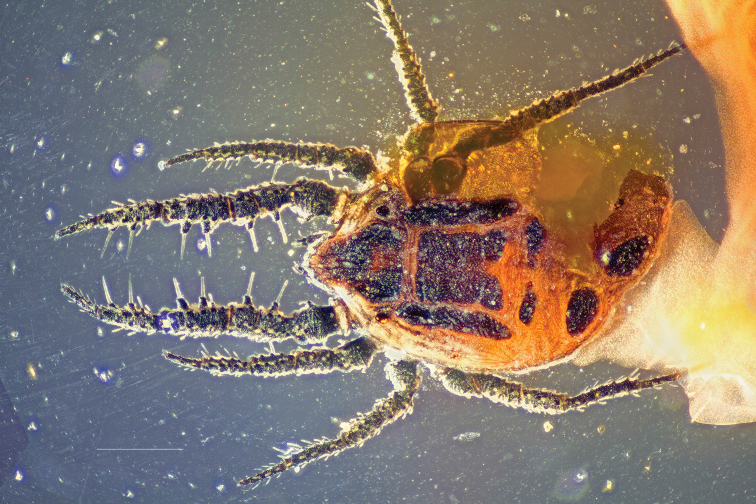
*Caeculus cremnicolus*, female. Micrograph of dorsal idiosoma, collected and slide mounted in 1978 by E.E. Lindquist. Scale bar: 0.5 mm.

### Phylogenetic analysis

**Figure 6.**
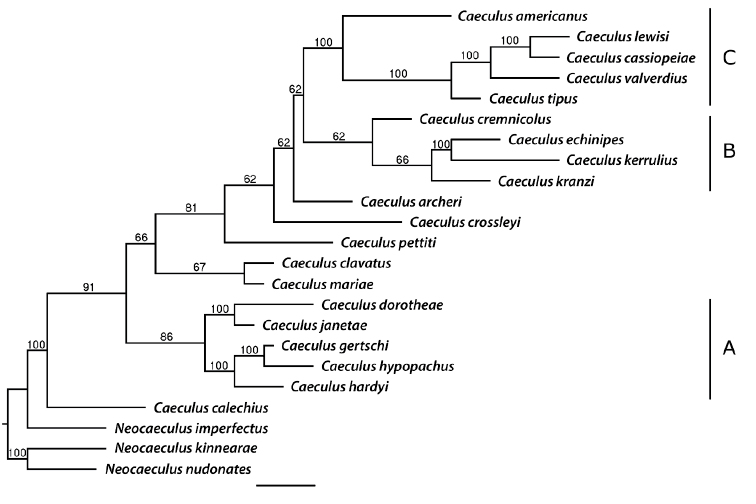
Phylogenetic 50% majority-rule consensus tree of *Caeculus* species based on character state matrix. Bootstrap values for 5000 replicates are above branches. Scale bar: 6.0 substitutions per phenotypic character.

The character state matrix contains 51 characters (Table 2), of which 27 are binary and the remainder occur in multiple states. The first 50 characters contributed to the phylogenetic analysis, and the last is a body length character that we retained only to improve the utility of the matrix as an identification tool but not used in the phylogeny. Among the characters used for phylogenetics, 88% were parsimony-informative. PAUP* assessed 5.659 × 10^9^ arrangements in 5000 replicates for the cladistic analysis, generating a 50% majority-rule consensus tree from 722 retained trees (Fig. 6). The parsimony score of the best tree was 199.

**Table 2.** Character states of *Caeculus* species (females) used for morphological comparison and cladistic analysis. Row number aligns to column number in Table 1. A setal pair refers to two setae in symmetry on either side of the mid-sagittal plane. An excluded seta is denoted by “excl.” Size character is excluded from analysis.

Character	States
1. Distribution	0 = Old World, 1 = SE North America, 2 = SW North America, 3 = N Central North America, 4 = NW North America, 5 = Western Australia
2. Aspidosomal Pa setae	0 = absent, 1 = 1 pair, 2 = 2 pairs, 3 = 3 pairs
3. Aspidosomal Pm setae	0 = absent, 1 = 1 pair, 2 = 2 pairs
4. Aspidosomal Pp setae	0 = 1 pair, 1 = 2 pairs, 2 = 3 pairs, 3 = 5 pairs
5. Anterior margin of aspidosoma acuminate	0 = no, 1 = yes
6. Anterior margin of aspidosoma notched	0 = no, 1 = yes
7. Centrodorsal opisthosoma a setae (excl. as)	0 = 1 pair, 1 = 2 pairs
8. Unpaired medial as seta present	0 = no, 1 = yes
9. Centrodorsal opisthosoma b setae (excl. bs)	0 = 1 pair, 1 = 2 pairs, 2 = 3 pairs
10. Unpaired medial bs seta present	0 = no, 1 = yes
11. Centrodorsal opisthosoma c setae (excl. cs)	0 = 1 pair, 1 = 2 pairs
12. Unpaired medial cs seta present	0 = no, 1 = yes
13. Laterodorsal opisthosoma a setae	0 = 1 seta, 1 = 2 setae, 2 = 3 setae
14. Laterodorsal opisthosoma b setae	0 = 1 seta, 1 = 2 setae , 2 = 3 setae
15. Laterodorsal opisthosoma c setae	0 = 1 seta, 1 = 2 setae, 2 = 3 setae
16. Mediodorsal opisthosomal sclerites fused	0 = no, 1 = yes
17. Mediodorsal opisthosomal d setae (excl. ds)	0 = 1 pair, 1 = 2 pairs, 2 = 3 pairs, 3 = 4 pairs
18. Unpaired medial ds seta present	0 = no, 1 = yes
19. Posterior opisthosomal e setae (excl. es)	0 = 1 pair, 1 = 2 pairs, 2 = 3 pairs, 3 = 4 pairs, 4 = 5 pairs, 5 = 8 pairs
20. Unpaired medial es seta present	0 = no, 1 = yes
21. Pluriposterior sclerite h setae (excl. hs)	0 = absent, 1 = 1 pair, 2 = 2 pairs
22. Unpaired medial hs seta present	0 = no, 1 = yes
23. Aggenital + ventral sclerite setae	0 = 2 pairs, 1 = 4 pairs, 2 = 6 pairs, 3 = 8 pairs, 4 = 9 pairs, 5 = 10 pairs, 6 = 12 pairs
24. Progenital valve setae	0 = 3 pairs, 1 = 4 pairs, 2 = 5 pairs, 3 = 6 pairs, 4 = 7 pairs, 5 = 8 pairs
25. Adanal setae	0 = absent, 1 = 1 pair, 2 = 2 pairs, 3 = 3 pairs
26. Pseudanal Ps setae	0 = 2 pairs, 1 = 3 pairs, 2 = 4 pairs
27. Unpaired medial seta posterior to anus	0 = no, 1 = yes
28. Trochanter I anterolateral setae	0 = 1 seta, 1 = 2 setae, 2 = 3 setae, 3 = 4 setae
29. Trochanter I anterolateral setal shape	0 = clavate, 1 = spiniform
30. Posterior/dorsal trochanter I setae	0 = 1 seta, 1 = 2 setae, 2 = 3 setae, 3 = 4 setae, 4 = 5 setae, 5 = 8 setae
31. Basifemur I anteroventral rake setal shape	0 = spiniform, 1 = subclavate
32. Telofemur I anteroventral rake setae	0 = 1 seta, 1 = 2 setae
33. Telofemur I anteroventral rake setal shape	0 = spiniform, 1 = subclavate
34. Femur I posteroventral rake setae	0 = absent, 1 = 1 seta
35. Genu I anteroventral rake/spiniform setae	0 = 1 seta, 1 = 2 setae, 2 = 3 setae, 3 = 5 setae
36. Genu I posteroventral rake setae	0 = absent, 1 = 1 seta, 2 = 2 setae, 3 = 3 setae, 4 = 4 setae
37. Tibia I anteroventral rake/spiniform setae	0 = 2 setae, 1 = 3 setae, 2 = 4 setae, 3 = 5 setae, 4 = 6 setae
38. Tibia I posteroventral rake/spiniform setae	0 = absent, 1 = 1 seta, 2 = 2 setae, 3 = 3 setae, 4 = 4 setae, 5 = 5 setae
39. Tarsus I anterior rake-like setae	0 = absent, 1 = 3 setae, 2 = 4 setae, 3 = 5 setae
40. Trochanter III anterolateral setae	0 = absent, 1 = 1 seta, 2 = 2 setae, 3 = 3 setae
41. Trochanter III anterolateral setal shape	0 = clavate, 1 = spiniform
42. Posterior/dorsal trochanter III setae	0 = 1 seta, 1 = 2 setae, 2 = 3 setae, 3 = 4 setae
43. Bothridial bt seta on tarsus I	0 = no, 1 = yes
44. Bothridial bt seta on tarsus II	0 = no, 1 = yes
45. Bothridial bt seta on tarsus III	0 = no, 1 = yes
46. Bothridial bt seta on tarsus IV	0 = no, 1 = yes
47. Dark sclerites on dorsal idiosoma in adults	0 = no, 1 = yes
48. Body encrusted with cemented particles	0 = no, 1 = yes
49. Tarsal claws unequal in size	0 = no, 1 = yes
50. Aspidosoma projecting anteriorly over gnathosoma in lateral view	0 = no, 1 = yes
51. Body length (mm)	0 = ≤ 0.90, 1 = 0.91 – 1.29, 2 = 1.30 – 1.59, 3 = 1.60 – 2.00

The phylogeny reveals three morphological clades, termed A, B, and C (Fig. 6). *Caeculus calechius* Mulaik is basal within the genus; this taxon and a few other species fall out independently from the clades. Clade A characterizes *C. dorotheae* Mulaik as sister to *C. janetae* Higgins & Mulaik (100%), and *C. gertschi* Mulaik sister to *C. hypopachus* Mulaik (100%). Despite lower branch support for the B clade, there is high support for *C. kerrulius* Mulaik being sister to *C. echinipes* (100%). Sister to clade B is the strongly supported C clade, which represents *C. lewisi* McDaniel & Boe as sister to *C. cassiopeiae* sp. nov. (100%), and this pair is sister to *C. valverdius* Mulaik (100%).

## Discussion

### Taxonomy

Although *Caeculus cassiopeiae* sp. nov. is morphologically most similar to *C. lewisi* McDaniel & Boe and *C. valverdius* Mulaik, several noteworthy differences exist (Table 1). The dichotomous key below lists six traits that distinguish the new species from *C. lewisi*. Additionally, following Franz (1952) and McDaniel and Boe (1990), the new species keys to *C. valverdius*, yet several traits separate them as well: (*i*) *C. valverdius* bears six *b* setae on the centrodorsal opisthosomal sclerite, but five *b* setae are arranged in an M-shape in the new species; (*ii*) each laterodorsal opisthosomal sclerite has a single *c* seta in *C. valverdius* whereas the new species has two *c* setae; (*iii*) the posterior opisthosomal sclerite has five *e* setae in *C. valverdius* whereas the new species has seven; (*iv*) *C. valverdius* has six setae on each progenital valve whereas the new species has seven; (*v*) the adanal sclerites each have three setae in *C. valverdius* and two in the new species; (*vi*) genu I bears a single posteroventral rake seta in the new species whereas *C. valverdius* has four; and (*vii*) *C. valverdius* has two anterolateral setae on the proximal half of trochanter III, but the new species has three that are more evenly distributed along the length.

### Phylogenetic analysis

A few apomorphies denote the relationships within clade A; *C. dorotheae* and *C. janetae* both have six anteroventral rake setae on tibia I, which is a unique character. *Caeculus gertschi* and *C. hypopachus* each bear four rake setae in that location, a character shared with some members of clade B. Of the species in clade A, most are not recorded as having tarsal bothridial setae *bt*, which occur in most other *Caeculus* as well as in the outgroups, indicating possible plesiomorphy for the rest of the genus. One exception is *C. gertschi* (Mulaik 1945). *Caeculus pettiti* Nevin and *C. mariae* Higgins & Mulaik also lack this trait, possibly resulting from homoplasy.

Aside from *C. krantzi* Coineau, the members of clade B exhibit dark dorsal idiosomal sclerites in adults, although this trait may be homoplastic as it recurs sporadically in the other clades and some taxa not in clades.

All members of the C clade possess five anteroventral rake setae on tibia I, except *C. tipus* Mulaik, which has four. However, McDaniel and Boe (1990) also describe a male and a nymph *C. lewisi* with four rake setae in this position. The species of clade C furthermore have four posteroventral rake setae on tibia I, although there are two such rake setae on *C. americanus*, basal in the clade. Rake seta number is regarded as more stable than other traits (Coineau 1974), so the above traits may be autapomorphies for the clade. Another potential apomorphy for clade C is two or three pairs of aspidosomal *Pa* setae, although Mulaik (1945) also describes *C. dorotheae* with three pairs of *PA*. All other congeners and all outgroup taxa have a single pair of *Pa* setae, which likely describes the ancestral state for the genus. Additionally, clade C species all have four to six centrodorsal opisthosomal *b* setae, also present in *C. crossleyi* Hagan and *C. pettiti*, so this may represent synapomorphy. The scarcity of autapomorphies in the topology likely reflects the variable nature of many traits in Caeculidae (Grandjean 1944; Coineau 1974).

### Key to adult *Caeculus* species (females)

**Table:** 

1	Aspidosoma with 0 or 1 pair of *Pa* setae on anterior margin, or if more *Pa* setae then only 1 pair of *Pp* setae near posterior margin	**2**
–	Aspidosoma with ≥ 2 pairs of *Pa* setae on anterior margin, and ≥ 2 pairs of *Pp* setae near posterior margin	**16**
2	Centrodorsal opisthosomal sclerite with 1 pair of setae at each the anterior margin (*a*), middle (*b*), and posterior margin (*c*), and with no unpaired medial setae; each laterodorsal opisthosomal sclerite with 1–2 *a* setae near the anterior margin, 1 *b* seta at middle, and 1 *c* seta near posterior margin; femoral segments of leg I with spiniform (never subclavate) rake setae	**3**
–	Not with above combination of characters	**8**
3	Trochanter I with 1 seta on both anterolateral and posterior/dorsal surfaces	***C. calechius* Mulaik**
–	Trochanter I with > 1 seta on anterolateral surface, or if 1 anterolateral seta, then with 2 posterodorsal setae	**4**
4	Tibia I with 6 anteroventral rake/spiniform setae	**5**
–	Tibia I with fewer anteroventral rake/spiniform setae	**6**
5	Trochanter I with 1 anterolateral seta; genu I with 5 anteroventral rake/spiniform setae; body not coated with cemented debris	***C. janetae* Higgins & Mulaik**
–	Trochanter I with 3 anterolateral setae; genu I with 3 anteroventral rake/spiniform setae; body encrusted with cemented particles	***C. dorotheae* Mulaik**
6	Tibia I with 3 anteroventral rake/spiniform setae; trochanter I anterolateral setae are clavate	***C. hardyi* Mulaik & Allred**
–	Tibia I with 4 anteroventral rake/spiniform setae; trochanter I anterolateral setae are spiniform	**7**
7	Anterior margin of aspidosoma acuminate; dark idiosomal sclerites	***C. hypopachus* Mulaik**
–	Anterior margin of aspidosoma not acuminate; pale idiosomal sclerites	***C. gertschi* Mulaik**
8	Body length ≤ 0.90 mm	**9**
–	Body length > 0.90 mm	**10**
9	Elongated bothridial setae on each tarsus; tibia I with 2 anteroventral rake/spiniform setae; dark idiosomal sclerites	***C. crossleyi* Hagan**
–	No elongated bothridial setae on any tarsus; tibia I with 3 anteroventral rake/spiniform setae; pale idiosomal sclerites	***C. mariae* Higgins & Mulaik**
10	Basi- and telofemur I with subclavate rake setae	***C. clavatus* Banks**
–	Basi- and telofemur I with spiniform rake setae	**11**
11	Mediodorsal opisthosomal sclerite with 8–9 setae	***C. pettiti* Nevin**
–	Mediodorsal opisthosomal sclerite with fewer setae	**12**
12	Pale idiosomal sclerites	**13**
–	Dark idiosomal sclerites	**14**
13	Posterior opisthosomal sclerite with 6 setae	***C. archeri* Mulaik**
–	Posterior opisthosomal sclerite with 11 setae	***C. krantzi* Coineau**
14	Tibia I with 2 anteroventral rake/spiniform setae; trochanter III with 3 anterolateral setae; pluriposterior sclerite with 1 unpaired medial *h* seta	***C. cremnicolus* Enns (Fig. 5)**
–	Tibia I with 3 anteroventral rake/spiniform setae; trochanter III with 1 anterolateral seta; pluriposterior sclerite with 1 pair of *h* setae and no unpaired medial seta	**15**
15	Aspidosomal anterior margin notched and lacking *Pa* setae; anterolateral surface of trochanter III with 1 spiniform seta; mediodorsal opisthosomal sclerites not fused; elongated bothridial setae on tarsi III and IV	***C. kerrulius* Mulaik**
–	Aspidosomal anterior margin not notched and bearing 1 pair of *Pa* setae; anterolateral surface of trochanter III with 1 clavate seta; mediodorsal opisthosomal sclerites fused; elongated bothridial setae present on all tarsi	***C. echinipes* Dufour**
16	Dark idiosomal sclerites; tibia I with 2 posteroventral rake/spiniform setae	***C. americanus* Banks**
–	Pale idiosomal sclerites; tibia I with 4 posteroventral rake/spiniform setae	**17**
17	Trochanter I with 2 anterolateral setae; progenital valves each with ≤ 5 setae	***C. tipus* Mulaik**
–	Trochanter I with 3 anterolateral setae; progenital valves with more setae	**18**
18	Genu I with 4 posteroventral rake setae; adanal sclerites each with 3 setae	***C. valverdius* Mulaik**
–	Genu I with 0 or 1 posteroventral rake seta; adanal sclerites each with 2 setae	**19**
19	Aspidosomal anterior margin with 2 pairs of *Pa* setae; basifemur I with 1 posteroventral rake seta; trochanter III with 3 anterolateral setae; centrodorsal opisthosomal sclerite with 5 *b* setae arranged in an “M”; laterodorsal opisthosomal sclerite with 2 *a* setae; posterior opisthosomal sclerite with 7 setae	***C. cassiopeiae* Bernard & Lumley, sp. nov. (Figs 1–4)**
–	Aspidosomal anterior margin with 3 pairs of *Pa* setae; basifemur I without posteroventral rake setae; trochanter III without anterolateral setae; centrodorsal opisthosomal sclerite with 3–4 *b* setae; laterodorsal opisthosomal sclerite with 1 *a* seta; posterior opisthosomal sclerite with 9 setae	***C. lewisi* McDaniel & Boe**

### Geographical distribution

The complete distribution ranges for the taxa described above are unknown, but the new find of *C. cassiopeiae* sp. nov. is 2074 km from the nearest recorded occurrence of *C. valverdius* in Los Lunas, New Mexico (Mulaik and Allred 1954), and 805 km from the record of *C. lewisi* near Newell, South Dakota (McDaniel and Boe 1990). A comparison of climates reveals that Los Lunas has a mean annual precipitation of 249.7 mm and a mean annual temperature of 13.45 °C for the 1981–2010 period, whereas data for Newell are 447 mm and 8.2 °C respectively (NCEI 2017). This gives Los Lunas a Köppen-Geiger climate classification of cold semi-arid steppe (*BSk*) like Medicine Hat, whereas Newell has a humid continental climate (*Dfa*) (Peel et al. 2007). The soil in both Los Lunas and Newell is brown chernozem/calcic aridisol (NRCS 2017, NSBD 2017), somewhat similar to the brown chernozem/ustic mollisol soil at Medicine Hat. These data show that although the new species is morphologically distinct from *C. lewisi* and *C. valverdius*, it shares climatic preferences with *C. valverdius*, and has a comparable soil habitat with both *C. lewisi* and *C. valverdius*.

The Albertan *C. cremnicolus* are 1911 km from the closest published location in Easley, Missouri (Enns 1958). The localities in Missouri have a humid subtropical climate (*Cfa*) by the Köppen-Geiger system, but the climates in the Alberta locations are incongruent; Writing-on-Stone Provincial Park is classed as cold semi-arid (*BSk*) yet Waterton Lakes National Park is continental subarctic (*Dfc*) (Peel et al. 2007). Soils among these sites are also variable. Along the Missouri River, sites have soils classified as gleyed regosol/fluvent entisol, and at Devil’s Backbone the soil is gray brown luvisol/udalfic alfisol (NRCS 2017). In Canada, the soil at Writing-on-Stone Provincial Park is a regosol/fluvent entisol similar to Missouri, but Waterton Lakes National Park has substrate that is classed as dystric brunisol/cryept inceptisol (NSBD 2017). These disparate abiotic conditions indicate that *C. cremnicolus* is a habitat generalist.

As the localities for *C. cremnicolus* and *C. cassiopeiae* sp. nov. are 126 km apart and in the same climate, their ranges could potentially overlap if *C. cremnicolus* can inhabit the soil at Medicine Hat. These species are nevertheless in separate clades (Fig. 6), signifying multiple introductions of the family into Canada. Such a pattern of introductions may be attributable to postglacial changes in biome distributions, which enabled fragmented xeric populations to expand northward from arid refugia after the last glacial maximum 18,000 years ago (Riddle and Hafner 2006; Graham et al. 2013). This possibility is intriguing considering that at the time Medicine Hat was near the southern limit of the Laurentide Ice Sheet, and by 14,000 years ago it was the first area in Canada freed of the ice as it became semi-arid grassland (Dyke 2005). Likewise, Thaler et al. (1993) described a new locality of *Microcaeculus austriacus* in Austria that was ~440 km from its known locations in the easternmost extent of the Alps, and alluded to postglacial warming as a possible explanation for the scattered distribution.

Our phylogenetic analysis suggests that the common ancestor of *Caeculus* inhabited southwestern North America, based on the known locations for *C. calechius* and Clade A (the most basal clade). The other clades also contain representatives from the North American southwest as well as those from other areas, and there is a diversity of locations represented by the taxa that are not in clades. *Caeculus echinipes* is the only species of *Caeculus* described to date from Europe. The results suggest that the ancestor of *C. echinipes* spread to Europe from North America. Further work to include molecular data would be helpful to clarify the weaker branch support shown in the topology.

## Conclusions

Our description of *Caeculus cassiopeiae* sp. nov. elevates the number of known Canadian caeculids to two. Based on a maximum parsimony analysis of a character state matrix for all members of the genus, it is most closely related to *C. lewisi*. The phylogeny also suggests multiple introductions of caeculids into Canada, and that the origin of the genus is the American southwest. Further collection of caeculids in North America is required to determine to what extent ranges overlap, examine ecology or additional morphological traits (e.g., minute sensory structures) that were not described in previously published taxonomic accounts, and to enable genetic analysis. Population studies can further describe the degree of intraspecific variation and clarify species boundaries. Our phylogeny provides the first analysis of the genus, which can be useful for future systematic studies that integrate taxonomy and genetics to develop a better understanding of the genus *Caeculus* and its family.

## Supplementary Material

XML Treatment for Caeculus cassiopeiae

XML Treatment for Caeculus cremnicolus
